# Fast Streamline Search: An Exact Technique for Diffusion MRI Tractography

**DOI:** 10.1007/s12021-022-09590-7

**Published:** 2022-06-18

**Authors:** Etienne St-Onge, Eleftherios Garyfallidis, D. Louis Collins

**Affiliations:** 1 NeuroImaging and Surgical Technologies Laboratory (NIST), Montreal Neurological Institute (MNI), Department of Neurology and Neurosurgery, McGill University, Montreal, QC Canada; 2 Luddy School of Informatics, Computing and Engineering, Indiana University, Bloomington, USA

**Keywords:** Tractography, Streamline, Polyline, White matter bundle, Binary search tree, Clustering

## Abstract

In this work, a hierarchical search algorithm is proposed to efficiently compute the distance between similar tractography streamlines. This hierarchical framework offers an upper bound and a lower bound for the point-wise distance between two streamlines, which guarantees the validity of a proximity search. The proposed streamline representation enables the use of space-partitioning search trees to increase the tractography clustering speed without reducing its accuracy. The resulting approach enables a fast reconstruction a sparse distance matrix between two sets of streamlines, for all similar streamlines within a given radius. Alongside a white matter atlas, this *fast streamline search* can be used for accurate and reproducible tractogram clustering.

## Introduction

In classical anatomy, the study of white matter fascicles and bundles connecting different brain regions required dissection. The non-invasive analysis of these connections has been greatly facilitated by the use of diffusion weighted MRI (Catani et al., 2002; Jones, 2008). From diffusion weighted MRI, tractography algorithms can be employed to investigate the white matter structure and connectivity (Wakana et al., 2007; Descoteaux, 2015; Jbabdi & Johansen-Berg, 2011). Representing white matter pathways, these tractography streamlines are often grouped in bundles for further analysis, such as tractometry (Bells et al., 2011; Chamberland et al., 2019a; Chamberland et al., 2019b).

Streamlines reconstructed from a tractography algorithm are composed of an ordered list of points, depicting local white matter position and trajectory. Each streamline is a polygonal chain, a set of connected line segments (also named polyline in computer graphics), with some specific characteristics that depend on the tractography algorithm. For example, most tractography algorithms reconstruct streamlines with a fixed step size (segment length) and a maximum turning angle (Tournier et al., 2012; Côté et al., 2013; Behrens et al., 2014).

Multiple tractography applications require a grouping of similar streamlines for analysis. These streamlines can be clustered based on shape similarity and proximity. Numerous algorithms and definitions of distance have been studied to improve the accuracy and efficiency of streamlines clustering (Guevara et al., 2011; Siless et al., 2013; Garyfallidis et al., 2012; Garyfallidis et al., 2016; Olivetti et al., 2017; Vázquez et al., 2020). Searching for the nearest streamline in a pre-segmented set of streamlines (called a bundle atlas) can be used to automatically dissect a tractogram into different bundles and white matter pathways (O’Donnell & Westin, 2007; Garyfallidis et al., 2018; Wang & Shi, 2019; Bertò et al., 2021). However, current approaches rely on space embedding techniques or subsampling without any distance-preserving guaranties, resulting in approximate distance.

In parallel, similar proximity search algorithms have been proposed in the data-mining field to analyze and compare time series data (Liao, 2005; Fu, 2011; Wang et al., 2013; Kotsakos et al., 2013). A multivariate time series could also be represented as a polygonal chain. Nonetheless, current distance measures for tractography streamlines do not directly fit in this framework. Interestingly, some bounded dimensionality reduction techniques employed for time series can be adapted to an existing streamline distance measure (Yi & Faloutsos, 2000; Keogh et al., 2001; Chan et al., 2003; Wang et al., 2013). One of these techniques, the piecewise aggregate approximation (Keogh et al., 2001), can be adapted to estimate Euclidean-based streamline distance, offering a lower bound which guarantees no false dismissal. In other words, this approximation never overestimates the given distance; thus, it never wrongly rejects streamlines in a radius search.

In this work, we focus on a streamline representation/simplification that conserves important distance properties. The resulting hierarchical representation enables the use of standard binary search trees to increase the clustering speed. In addition, the theoretical upper and lower bounds are used to ensure the accuracy of the proximity search. The resulting formulation can be applied to efficiently compute an exact nearest neighbor (NN) or k-nearest neighbors (KNN) search within a maximum distance.

## Methods

This section describes the proposed hierarchical approach to efficiently evaluate streamlines distance. For this, we first detail the mathematical framework to compute the distance between two streamlines. Followed by interesting mathematical properties used to optimize streamline representation and construct the proposed hierarchical search. Afterward, this approach is employed to search for the nearest streamline in a pre-segmented white matter bundle atlas, described in the "Experiments" section.

### Distance Between Two Streamlines

A tractography streamline $S=[\mathbf{s} _1,...,\mathbf{s} _m]$ is defined as an ordered series of *m* points, where each of those points lives in a three-dimensional space $\mathbf{s} _i \in \mathbb {R}^3$, $i \in \{1, ..., m\}$. The distance between two points in *n*-dimensions ($\mathbf{x} ,\mathbf{y} \in \mathbb {R}^n$) is generally defined by the Minkowski distance ($L^p$-norm).

1 $$\begin{aligned} \text {dist}_{L^p}(\mathbf{x} ,\mathbf{y} ) \mathrel {:=}|| \mathbf{x} - \mathbf{y} ||_p = \Big (\sum _{j=1}^n |x_j-y_j|^p\Big )^\frac{1}{p} \end{aligned}$$

This distance is a generalization of both the Manhattan ($L^1$) and the Euclidean ($L^2$) distance. It satisfies the triangle inequality for any $p \ge 1$, resulting in a valid metric. This can be extended to define the maximum norm ($L^\infty$) as $p \rightarrow \infty$, dual to the $L^1$ norm in finite-dimensional spaces. This research focuses on the $L^1$ and the $L^2$ norms. Nonetheless the $L^\infty$ provides some bounding capacity and is sometimes used in binary search trees.

2 $$\begin{aligned} \text {dist}_{L^\infty }(\mathbf{x} ,\mathbf{y} ) \mathrel {:=}\left\| \mathbf{x} - \mathbf{y} \right\| _\infty = \max _{j=1}^n \left( |x_j - y_j|\right) \end{aligned}$$

**Fig. 1:**
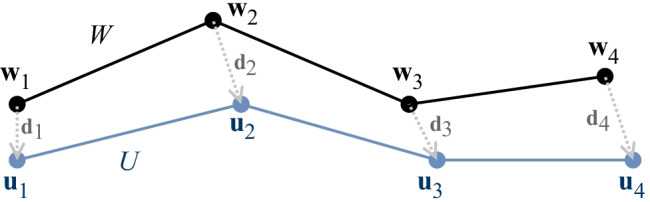
Pair-wise distance between two tractography streamlines (*U*, *W*) from an ordered list of points ($m=4$), where $\text {dist}_{L^p}(U,W) \mathrel {:=}\sum _{i=1}^m || \mathbf{u} _i - \mathbf{w} _i ||_p = \sum _{i=1}^m || \mathbf{d} _i ||_p \,$

The proposed method utilizes the sum (or average) of $L^p$-norm to compute the distance between two streamlines ($U=[\mathbf{u} _1,...,\mathbf{u} _m], W=[\mathbf{w} _1,...,\mathbf{w} _m]$).

3 $$\begin{aligned} \text {dist}_{L^p}(U,W)&\mathrel {:=}\sum _{i=1}^m || \mathbf{u} _i - \mathbf{w} _i ||_p = \sum _{i=1}^m \Big (\sum _{j=1}^n |u_{i,j}-w_{i,j}|^p\Big )^\frac{1}{p} \end{aligned}$$

4 $$\begin{aligned} \text {mdist}_{L^p}(U,W)&\mathrel {:=}\frac{1}{m} \, \text {dist}_{L^p}(U,W)\end{aligned}$$

5 $$\begin{aligned} \text {dist}_{\text {MDF}}(U,W)&\mathrel {:=}\min \big (\text {mdist}_{L^2}(U,W)\,,\,\, \text {mdist}_{L^2}(U,W')\big ) \end{aligned}$$

The “mdist$_{L^p}(\cdot ,\cdot )$” is employed to compute the average point-wise distance. This is done to normalize the distance by the number of points. When computed for both ascending ($W=[\mathbf{w} _1,...,\mathbf{w} _m]$) and descending ordered points ($W'=[\mathbf{w} _m,...,\mathbf{w} _1]$), the average $L^2$ distance is equivalent to the minimum-average direct flip (MDF) proposed by Garyfallidis et al. (2012). The minimum-average direct flip is often used for streamlines clustering, similarity search and registration (Olivetti et al., 2017; Garyfallidis et al., 2018). For tractography, each point is in a tridimensional space, but this measure could be used in higher dimensions. The “dist$_{L^p}(\cdot ,\cdot )$” between two streamlines is depicted in Fig. 1, equivalent to the sum of the norm of directed vectors ($\mathbf{d} _i = \mathbf{u} _i - \mathbf{w} _i$). This sum of the norm is also known as the $L^{p,1}$ entry-wise matrix norm.

### Sum of Norm Properties

In this subsection, a few interesting properties of the sum of $L^1$ and $L^2$ are described. These characteristics are used to modify the streamline representation while keeping important distance properties. These mathematical remarks are further detailed in Appendix A.

#### Remark 1

When using Manhattan ($L^1$) distance, comparing two lists composed of *m* *n*-dimensional points ($\mathbf{u} _i,\mathbf{w} _i \in \mathbb {R}^n, \, i \in \{1, ..., m\}$) is equivalent to computing the $L^1$ distance between two $m \times n$ dimensional points ($\mathbf{u }, \mathbf{w } \in \mathbb {R}^{m \times n}$).

6 $$\begin{aligned} \text {dist}_{L^1}(U,W)&= \sum _{i=1}^m \left\| \mathbf{u} _i - \mathbf{w} _i \right\| _1 = \left\| \mathbf{u } - \mathbf{w } \right\| _1 \end{aligned}$$

#### Remark 2

The $L^1$ distance in *n*-dimensions can be used as an upper and a lower bound obtained from Hölder’s inequality.

7 $$\begin{aligned} \left\| \mathbf{x} \right\| _p \le \left\| \mathbf{x} \right\| _q \le n^{ (1/q - 1/p) } \left\| \mathbf{x} \right\| _p \,,\,\, \text {for} \,\, 0< q < p \end{aligned}$$

**Fig. 2:**
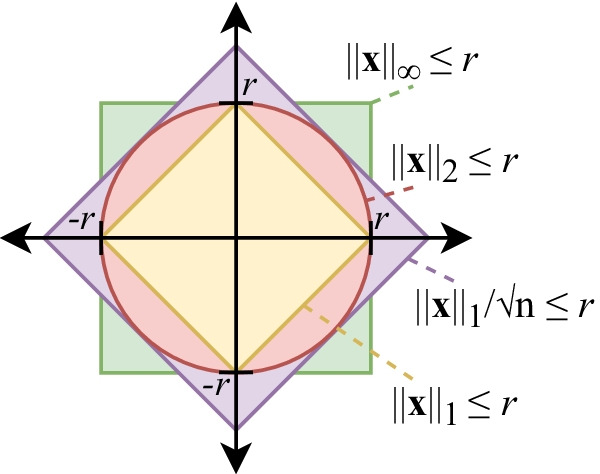
Illustration of norm inequality from *remark* 2 (Eq. 8). In 2D, the Euclidean distance ($L^2$), displayed in red, is bounded between ${L^1}/\sqrt{2}$ and $L^1$

From this general equation, the Euclidean ($L^2$) distance can be bounded with $L^1$ with the previous inequality ($q=1, p=2$). Figure 2 illustrates this inequality with bounded volume ($\left\| \mathbf{x} \right\| _p \le r$).

8 $$\begin{aligned} \frac{1}{\sqrt{n}}\left\| \mathbf{x} \right\| _1 \le \left\| \mathbf{x} \right\| _2 \le \left\| \mathbf{x} \right\| _1 \le \sqrt{n} \left\| \mathbf{x} \right\| _2 \end{aligned}$$

The sum of distances (p-norm) follow the same rules, since all summed values are positive.

9 $$\begin{aligned} \frac{1}{\sqrt{n}} \sum _{i=1}^m \left\| \mathbf{u} _i - \mathbf{w} _i \right\| _1&\le \sum _{i=1}^m \left\| \mathbf{u} _i - \mathbf{w} _i\right\| _2 \le \sum _{i=1}^m \left\| \mathbf{u} _i - \mathbf{w} _i \right\| _1 \end{aligned}$$

#### Remark 3

If $\overline{\mathbf{u }} = \frac{1}{m} \sum _{i=1}^m \mathbf{u} _i$ is the mean position of a streamline, then the distance between the mean position of two streamlines is always smaller or equal to the average point-wise distance.

10 $$\begin{aligned} \text {dist}_{L^p}(\overline{\mathbf{u }}, \overline{\mathbf{w }}) = \big \Vert \overline{\mathbf{u }} - \overline{\mathbf{w }} \big \Vert _p \,\le&\, \frac{1}{m} \sum _{i=1}^m \big \Vert \mathbf{u} _i - \mathbf{w} _i \big \Vert _p = \text {mdist}_{L^p}(U,W) \end{aligned}$$

Thus, averaging points together can be used to reduce the number of points to compare, without increasing the distance. This type of aggregation of points is used extensively for time series analysis, such as the piecewise aggregate approximation (Keogh et al., 2001), or Haar wavelet transform (Chan et al., 2003).

### Streamlines Representation & Simplification

#### Resampling

**Fig. 3:**
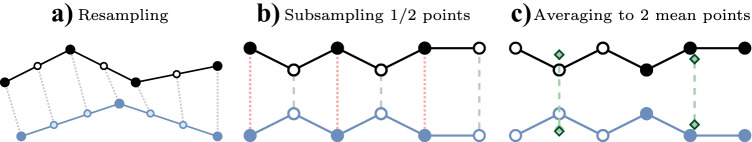
**a** Uniformly resampling streamlines can be used to make two streamlines, with unequal number of points, directly comparable using a point-wise distance; for equal length segments, this can be computed with the least common multiple of the number of segments. **b** Subsampling points can reduce (gray dashed lines) or increase (red dotted lines) the average point-wise distance between two streamlines. **c** Averaging points (green dashed lines) never increases this distance - it can only reduce it

Some form of resampling is required when comparing streamlines with different numbers of points with a point-wise distance. Tractography generates streamlines with a fixed step size, resulting in individual segments of equal length. Thus, to compare streamlines from start to end, each segment can be subdivided according to the least common multiple of the number of segments. This subdivision ensure an uniform distribution of points along each streamline for the point-wise distance without changing its geometry (see Fig. 3-a).

#### Downsampling

Subsampling is often used to reduce streamline complexity, but in the general case, it does not offer any bounding property. Therefore, removing points before the comparison of streamlines can reduce or increase the average distance between them. This is illustrated in Fig. 3-b, where keeping the filled-in points will increase the distance, and keeping the hollow points will decrease it. Thus, some neighbors could be missed when doing a proximity search using subsampled streamlines, resulting in an approximate search.

#### Averaging

As demonstrated earlier in *remark* 3, averaging points together never increases the average distance (“mdist$_{L^p}(\cdot ,\cdot )$”). Therefore, when searching for all similar streamlines in a given radius (*r*) using averaged points, it is guaranteed that the distance remains inside that radius. Consequently, computing the barycenter, or multiple mean points (“sub-barycenters”), is an effective way to reduce the number of comparisons (i.e. to reduce dimensionality) for tractography streamline proximity search. This concept of aggregating points will be used to a generate a hierarchical comparison method, similar to a multiscale approach. Fig. 3-c illustrates this simplification by averaging together 3 points along each streamlines, resulting in 2 mean points to compare.

**Fig. 4:**
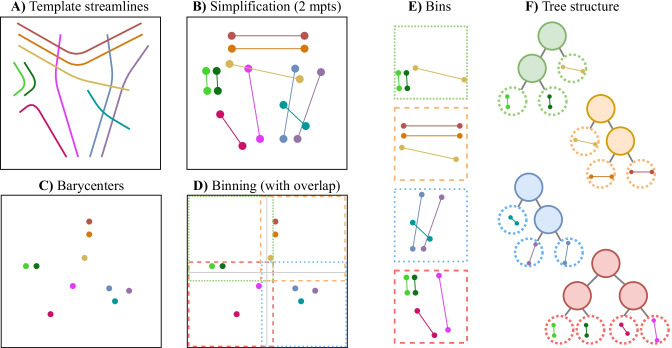
Template construction for the proposed hierarchical *fast streamline search*. **A** Streamlines in a given template, **B** template simplification by averaging resulting in $\mu$ mean points (mpts) per streamline, **C** barycenter (1 mean point) per streamline, **D**-**E** organizing streamlines using barycenter bins with an overlap greater or equal to the search radius, **F** space-partitioning tree structure for each bin using $\mu$ mean points

### Proposed Hierarchical Streamline Representation

**Fig. 5:**
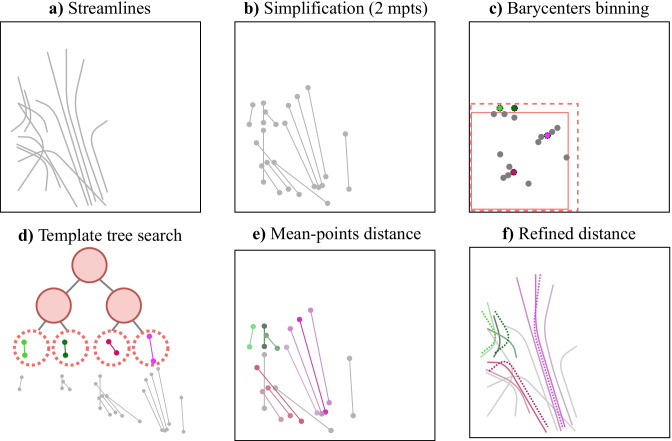
Streamlines nearest neighbor search to a previously generated template (see Fig. 4): **a** given streamlines to cluster, **b** simplification by averaging resulting in $\mu$ mean points (mpts) per streamline, **c** barycenter (1 mean point) per streamline, binned using template bins (without overlap), **d**-**e** for each given streamline compute the distance to template streamlines in the same bin within the search radius using $\mu$ mean points employing the space partitioning tree, **f** recompute the complete point-wise distance for all neighbors pair, given by the previous step, and return the nearest

Based on previous remarks, we propose a new hierarchical approach for tractography streamlines proximity search (exact NN or KNN) within a maximum distance. In this subsection, the framework is detailed in three procedures: *barycenter binning*, *simplification by averaging*, and *distance refinement*. When this approach is employed to search for the nearest streamline in a template (pre-segmented white matter bundle atlas), these procedures are used for both: the template construction (Fig. 4), and the resulting hierarchical streamlines search (Fig. 5).

#### Barycenter Binning

First, the barycenter of a streamline can be used as an initial proximity search. Because the distance between two barycenters is never greater than the “mdist$_{L^p}(\cdot ,\cdot )$” (see *remark* 3), it can be used to limit the proximity search (Fig. 5-c). Coordinates for all barycenters can be grouped together on a regular grid. When searching for all similar streamlines within a specified range (*r*), only the current grid and its neighbors (within distance *r*) need to be examined. The binning size can be optimized based on the amount of streamlines and the radius (*r*) of the proximity search; smaller bins will increase the preprocessing and construction time, but reduce the subsequent search time. Moreover, this barycenter binning provides independent bins, enabling efficient multithreading and the reduction of memory usage. Consequently, each bin can be separated and processed individually, only requiring the current and neighboring bins. When searching in a template, bins can be generated with an overlap to avoid to look at neighboring bins (see Fig. 4-D).

#### Simplification by Averaging 

Second, streamline points can be aggregated to create a simplified version with $\mu$ mean points (Fig. 5-b). This is done to reduce the number of dimensions when using a binary search tree, thereby “avoiding” the *curse of dimensionality* (Marimont and Shapiro, 1979; Verleysen and François, 2005; Pestov, 2013). When the number of mean points ($\mu$) is a divisor of the initial number of points (*m*) the *remark* 3 remains true; otherwise a continuous averaging needs to be done. Afterwards, each streamline’s mean points are vectorized in a $\mu \times n$ vector, to employ both *remarks* 1-2. This vectorization enables the use of standard space-partitioning tree structure, which greatly increase the search speed (see Fig. 5-d). For the distance between streamlines “mdist$_{L^p}(\cdot ,\cdot )$”, with the $L^2$ norm per points, the searching range need to be increased by the square root of the spatial dimensionality ($\sqrt{n}$). Since the distance between simplified streamlines using $\mu$ mean points is always smaller than or equal to the tractography streamline point-wise distance (*remark* 3), this aggregation enables the search for all similar streamlines within a certain radius, without missing any streamlines (Fig. 5-e). This results in a radius search with no false dismissals (no false negatives), where remaining false positives can be rectified with a refinement step.

#### Distance Refinement

Finally, the resulting similar streamlines with their respective distances, obtained from the proximity search using simplified streamlines, can be refined by computing the complete distance (without simplification). When searching for all similar streamlines within a radius (i.e. proximity search), streamlines with a complete distance larger than the desired radius need to be filtered out (Fig. 5-f). KNN search, within a maximum radius, can be done by computing refined distances and extracting the first *K* streamlines.

## Experiments

### Dataset

#### Tractography

For the evaluation, we utilized 44 subjects from the Human Connectome Project (HCP) dataset (Van Essen et al., 2013). Tractography streamlines were reconstructed using probabilistic *particle filtering tractography* (Girard et al., 2014) implemented in *Dipy* (Garyfallidis et al., 2014). Resulting streamlines were aligned to the MNI space (ICBM 2009a, Fonov et al., 2011) using ANTs affine registration (Avants et al., 2008). This registration was computed from the T1-weighted image of each subject (already aligned with the distortion corrected diffusion space) to the MNI template.

#### Bundle Atlas

The bundle atlas employed for the experiment is detailed in Garyfallidis et al. (2018); Yeh et al. (2018). Streamlines from this atlas were already aligned to the MNI space (ICBM 2009a, Fonov et al., 2011), which is composed of 33 bundles (9 inter-hemispheric, 12 intra-hemispheric) with a total of 210K streamlines.

#### Streamlines

All streamlines were defined with 32 points ($m=32$), to limit the variability in our testing, and make the proposed proximity search comparable to RecoBundles (Garyfallidis et al., 2018), because RecoBundles downsamples all streamlines to a fixed number of points (12). For the proposed approach, all streamlines from the atlas were compared with both ascending and descending (flip) order, resulting in 420K streamlines. This makes the employed distance “mdist$_{L^2}(\cdot ,\cdot )$” equivalent to RecoBundles’ minimum-average direct flip distance.

### Evaluation

The proposed hierarchical streamline search was quantitatively evaluated by measuring the computation time. Each streamline search method was computed twice per subject to avoid aberrant run time, keeping the smallest time for each subject (except for longer run without binning or without mean points). This computation time is afterward averaged over all 44 subjects to compare the efficiency of the proposed method with various parameters. This computation time did not include any file loading or saving time. The proposed approach was evaluated with and without the *barycenter binning* at various *bin_size* (4mm, 8mm, 12mm). The *simplification by averaging* was compared at different numbers of mean points (2, 4, 8). Without *barycenter binning* and *simplification*, this is equivalent to a brute force search with quadratic time.

For each subject, multiple sets of streamlines were used to vary the total amount of streamlines (500K, 1M, 2M, 4M). The proximity search radius was evaluated from 2mm to 12mm, in 2mm steps. The proximity search was applied to 44 subjects using all 33 bundles from the atlas.

The proposed algorithm was also compared to RecoBundles (Garyfallidis et al., 2018) with various number of streamlines. RecoBundles was run with its default parameters from *Scilpy*(v1.1.0): subsampling streamlines to 12 points, a pruning distance of 8mm, and a clustering threshold of 12mm (Garyfallidis et al., 2014; Garyfallidis et al., 2016; Rheault, 2020). It should be noted that the proposed *fast streamline search* is not equivalent to RecoBundles; RecoBundles subsamples streamlines and relies on the QuickBundles clustering algorithm, resulting in an approximate search. Moreover, RecoBundles/QuickBundles prune clusters using an adapted clustering threshold for each bundle. The goal of this evaluation is to give an idea of the clustering speed of the proposed streamline search method, compared to a state-of-the-art similarity-based clustering method (RecoBundles). Computation times were measured from a single core on Intel’s 2.4*GHz* Skylake 6148 processor. The proposed method employs *Scipy*(v1.6.3) cKDTree for space-partitioning (Virtanen et al., 2020).

Streamline simplification errors were evaluated to compare the conventional subsampling and the proposed mean points averaging. In addition, inaccuracy was estimated using false positive and false negative rates compared to an exact brute force search from 500K streamlines. For this accuracy test, RecoBundles results were averaged from 30 runs, using different random seed, to avoid outliers with this stochastic approach. All distance values and estimated errors are reported in millimeters.

## Results

### Computation Time

**Fig. 6:**
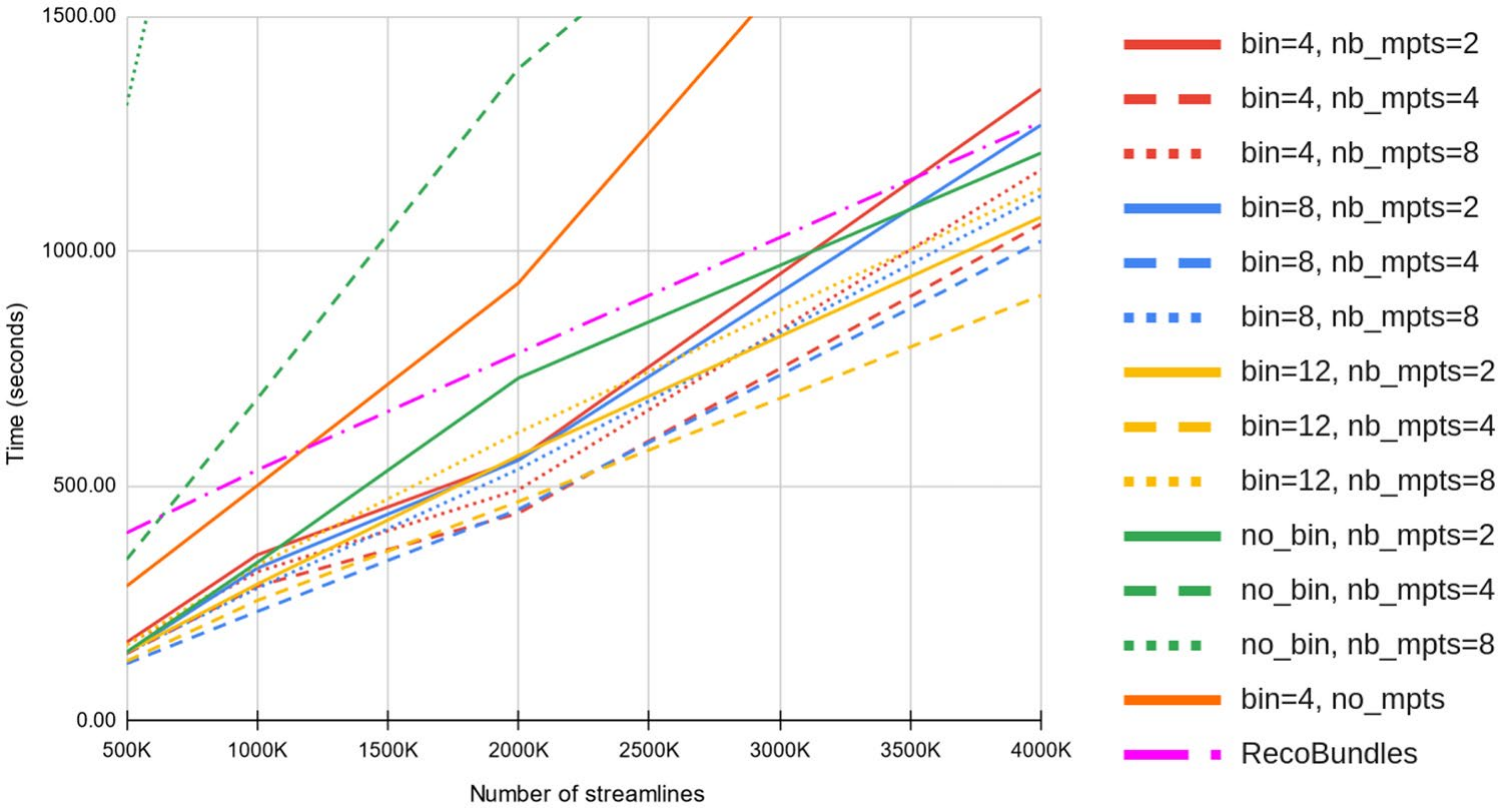
Streamlines proximity search ($L_2 \le 8mm$) time comparison with various parameters, for all 33 bundles in the atlas. Computation times were averaged over 44 subjects from the HCP dataset

**Fig. 7:**
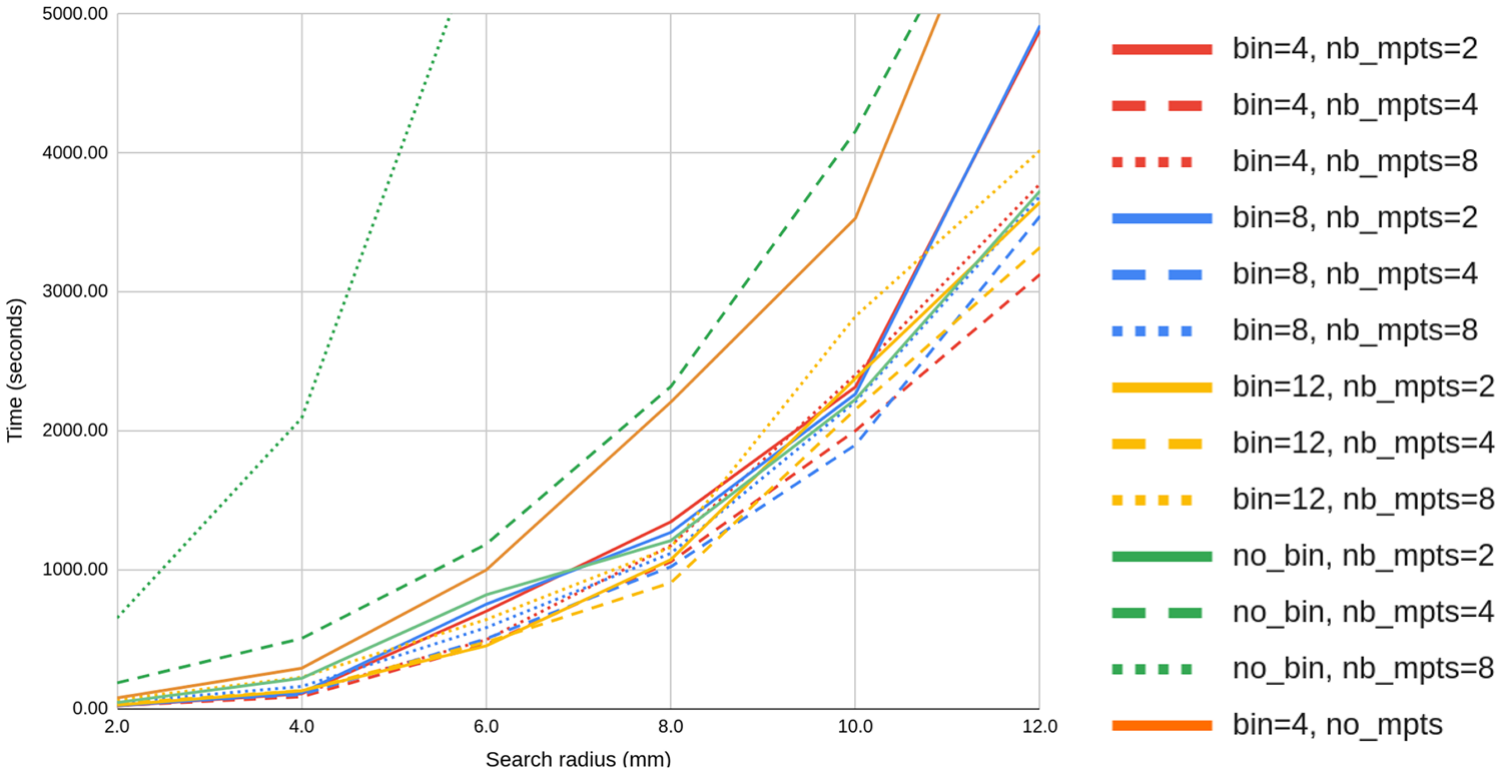
Clustering time comparison using 4 million streamlines for different search radii ($L_2 \le r$), for all 33 bundles in the atlas. Computation times were averaged over 44 subjects from the HCP dataset

Figure 6 details the computation time for streamline proximity search within 8*mm* (mdist$_{L^2}(\cdot ,\cdot ) \le 8mm$) at various numbers of streamlines (500K, 1M, 2M, 4M), searching for similar streamlines in a bundle atlas of 210K streamlines. When using both *barycenter binning* and *simplification*, the resulting clustering speed is comparable to RecoBundles. Figure 7 presents the computation time as a function of the search radius (from 2mm to 12mm) for 4 million streamlines. When using *barycenter binning*, a *simplification* with 4 mean points (nb_mpts=4) performs slightly better for any number of streamlines (from 500K to 4M) and search radius (from 2mm to 12mm). Using less mean points (nb_mpts=2) decrease the tree search time but increase even more the *distance refinement* computation time. This is reversed with more mean points (nb_mpts=8), since it reduces the *distance refinement* time but further increases the search time. It can be observe from Figs. 6, 7, that the optimal *barycenter binning* size varies in function of the number streamlines and search radius.

**Table 1:** Distance errors when using subsampling or mean points at various number of points (4, 8, 16). Comparing the estimated distance to the exact distance (with 32 points), from 500K streamlines to the left Corticospinal tract (CST) bundle in the atlas. The average error for each approach is presented with both mean absolute error (MAE) and mean squared error (MSE). The minimum and maximum differences are obtained from the exact distance minus the distance with resampled streamlines. All distance values and estimated errors are in millimeters

resampling	nb. points	MAE	MSE	min diff.	max diff.
subsample	16	0.7529	0.6730	-3.0529	1.4244
subsample	8	2.4034	6.7801	-9.3363	3.4591
subsample	4	6.4656	48.6004	-26.4175	12.9009
mean points	16	0.0360	0.0015	0.0006	0.5940
mean points	8	0.1773	0.0362	0.0019	2.7803
mean points	4	0.7210	0.6087	0.0043	8.1184

**Table 2:** Number of false positive (left) : false negative (right) using a brute force search, Recobundles and the proposed *fast streamline search* (FSS) without or with refinement. The 8mm radius search was done using 500K streamlines and the left Corticospinal tract (CST) atlas. Each method was compared with multiple number of points (4, 8, 16, 32), using both the conventional subsampling (normally used by Recobundles) and the proposed mean points simplification

resampling	nb. pts	brute force			Recobundles			FSS no refine			FSS refined		
none	32	0	:	0	0	:	1465	0	:	0	0	:	0
subsample	16	0	:	22	0	:	1465	0	:	22	0	:	22
subsample	8	0	:	75	4	:	1264	0	:	75	0	:	75
subsample	4	1	:	152	6	:	1346	1	:	152	0	:	152
mean points	16	2	:	0	0	:	1461	2	:	0	0	:	0
mean points	8	12	:	0	14	:	1181	12	:	0	0	:	0
mean points	4	40	:	0	18	:	1284	40	:	0	0	:	0

### Quantitative Comparison

Distance errors from downsampling streamlines are presented in Table 1. Overall, the proposed mean points results in a smaller mean absolute/squared error. Table 2 depicts the number of false positives and false negatives for each method at various resampling. Since the mean points approach did not increase the distance between two streamlines (from a positive minimum difference in Table 1), it resulted in zero false negatives when using a brute force approach. Without refinement, the *fast streamline search* is equivalent in accuracy to an exhaustive search with simplified streamlines. It can be noted that the proposed *fast streamline search* with refinement and mean points simplification results in an exact search, based on the “mdist$_{L^2}(\cdot ,\cdot )$” measure. Thus, only computation time varies when changing the bin size or the number of mean points, resulting distances and clustered streamlines do not change.

### Qualitative Comparison

**Fig. 8:**
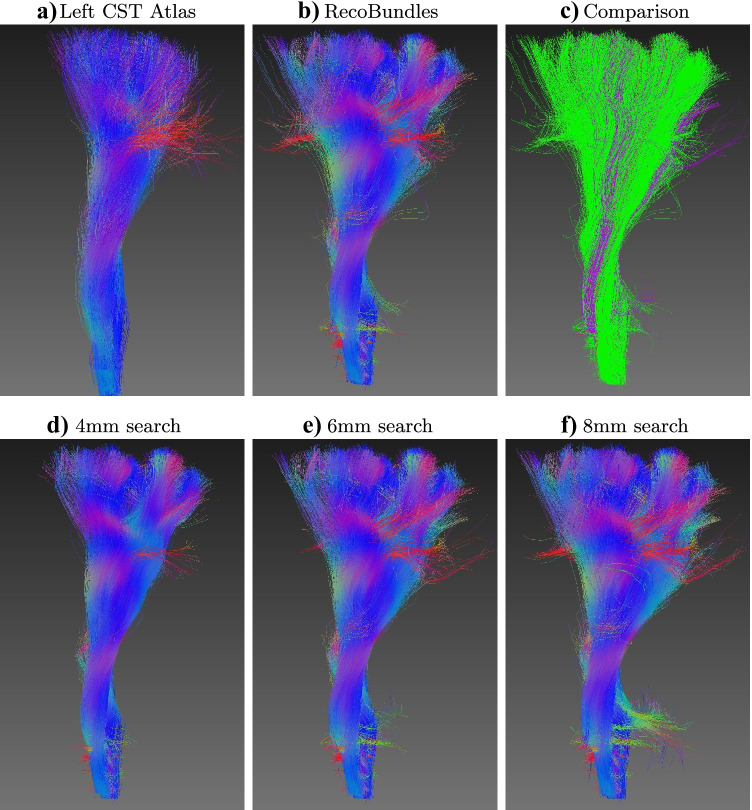
Results of the proximity search for the left Corticospinal tract (CST) from single HCP subject: **a** the bundle atlas from Garyfallidis et al. (2018); Yeh et al. (2018), **b** RecoBundles result, **c** RecoBundles result (in green) showing in purple streamlines missing in RecoBundles (false negatives) but present in the proposed technique with an exact search of 6mm radius. The proposed proximity search, mdist$_{L^2}(\cdot ,\cdot ) \le r$, using a radius of: **d** 4mm, **e** 6mm, and **f** 8mm. Streamlines are colored based on the local orientation (x,y,z to RGB) with the exception of c)

**Fig. 9:**
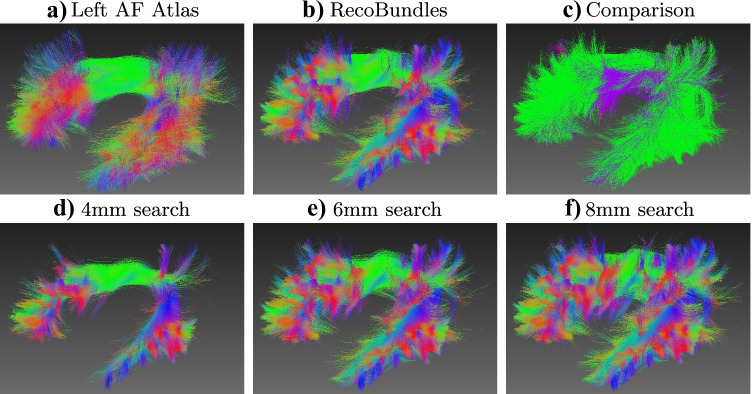
Results for the left Arcuate Fasciculus (AF): **a** the bundle atlas, **b** RecoBundles result, **c** RecoBundles result (in green) showing in purple streamlines missing in RecoBundles (false negatives) but present in the proposed technique with an exact search of 6mm. The proposed proximity search, mdist$_{L^2}(\cdot ,\cdot ) \le r$, using a radius of: **d** 4mm, **e** 6mm, and **f** 8mm

**Fig. 10:**
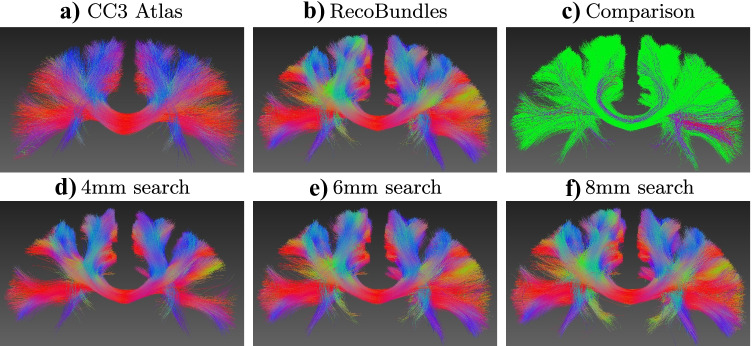
Results for the central portion of the Corpus Callosum (CC3): **a** the bundle atlas, **b** RecoBundles result, **c** RecoBundles result (in green) showing in red a few streamlines missing in RecoBundles (false negatives) but present in the proposed exact search at 4mm radius, and in purple, streamlines missing in RecoBundles but present in the proposed search at 6mm radius. The proposed proximity search, mdist$_{L^2}(\cdot ,\cdot ) \le r$, using a radius of: **d** 4mm, **e** 6mm, and **f** 8mm

**Fig. 11:**
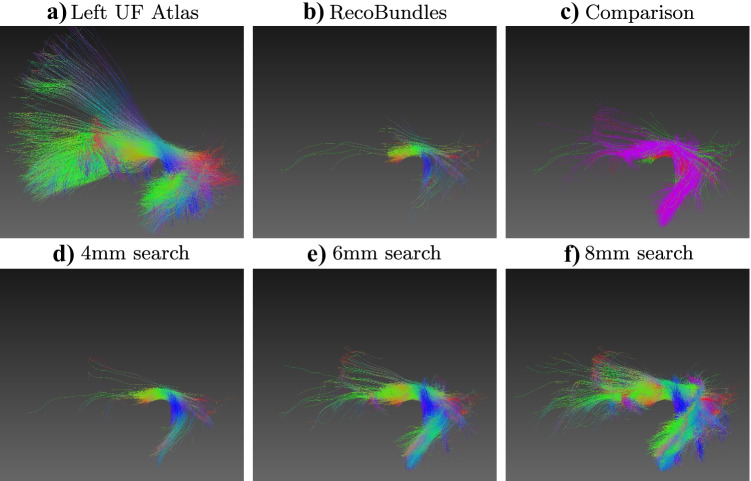
Results for the left Uncinate Fasciculus (UF): **a** the bundle atlas, **b** RecoBundles result, **c** RecoBundles result (in green) showing in red a few streamlines missing in RecoBundles (false negatives) but present in the proposed exact search at 4mm radius, and in purple, streamlines missing in RecoBundles but present in the proposed search at 6mm radius. The proposed proximity search, mdist$_{L^2}(\cdot ,\cdot ) \le r$, using a radius of: **d** 4mm, **e** 6mm, and **f** 8mm

Figure 8 shows streamlines extracted using both the proposed method (radius of 4mm, 6mm or 8mm) and RecoBundles. Both clusters were obtained from the Corticospinal tract (CST) in the bundle atlas. Results for other bundles are displayed in Appendix B (Figure 9, 10 and 11).

## Discussion

Overall, the proposed approach using 4 mean points results in the fastest computation time on average. Streamlines *simplification* with 4 mean points is a good trade-off between *distance refinement* computation time and tree search speed. In addition, the optimal *barycenter binning* size varies in function of the search radius, the number of streamlines and also from one subject to another. Nonetheless, not using this *barycenter binning* generally results in slower performance (green lines in Figs. 6 and 7) and is highly dependent of the bin size. Directly using all streamlines points (32), without *simplification* (orange line in Fig. 6), results in a poor computation time and heavy memory usage for the binary search tree. Bins of 8mm and 12mm without *simplification* are not displayed since they were significantly slower than binning at 4mm.

Depicted in Tables 1-2, the proposed framework (barycenter binning, simplification by averaging and distance refinement) accurately find all similar streamlines without any false positives or false negatives. Results are visually comparable (Fig. 8) to existing approaches that use an approximate similarity search (RecoBundles), where further comparison are displayed in Appendix B.

Despite this comparison, some part of this *fast streamlines search* algorithm could be directly integrated inside QuickBundles and RecoBundles to further improve their clustering speed when matching bundle centroids. Additionally, mean points could be employed in other tractography approaches, instead of subsampling, to reduce simplification errors when computing the distance between streamlines.

The proposed lower and upper bound definitions could be further improved using specific properties of tractography streamlines, such as the step size and maximum curving angle. However, these values would change from one tractography algorithm to another. As mentioned previously, tractography streamlines normally have a fixed segment length, however some researchers compress streamlines with the Ramer–Douglas–Peucker algorithm (Hershberger & Snoeyink, 1992) or a similar variant for tractography (Presseau et al., 2015) to save disk space. It should be noted, that compression algorithms modify streamlines thus they will change the original distance.

Other approaches could be used to further reduce the number of points (or dimensions) required when employing a search tree (O’Donnell & Westin, 2007; Olivetti et al., 2012; Wang & Shi, 2019; Legarreta et al., 2021). Nevertheless, those dimensionality reduction techniques on tractography streamlines do not preserve distances nor guarantee any lower/upper limits on distance, resulting in an approximate neighbor search.

### Applications

This proposed approach would be useful in a clinical setting when the search accuracy is critical, especially when missing streamlines (from false negatives) could significantly alter the analysis. This type of hierarchical search will become necessary when working with large tractograms, because computing an exhaustive search would be unfeasible. Still, before comparing streamlines, validating the tractography reconstruction and brain registration is crucial for clinical applications.

### Limitation & Future Work 

This proposed method and streamlines simplification were specifically designed for the sum (or average) of Minkowski p-norm. This hierarchical approach might be adaptable to other streamlines similarity measures described by Olivetti et al. (2017), however it would requires a redefined simplification algorithm along with new upper and lower bounds.

## Conclusion

The proposed framework efficiently and accurately search for all similar tractography streamlines inside a given radius. This method can be used to cluster streamlines into bundles, based on an given white matter atlas. The use of *simplification by averaging* (mean points) combined with a space-partitioning search tree significantly reduces the query time, with no false dismissals. Furthermore, *barycenter binning* provides independent bins, enabling efficient multithreading and the reduction of memory usage. Finally, this proposed method guarantees accurate results and is comparable in speed to existing approaches using approximate similarity search.

## Information Statement Sharing

The datasets employed in this experiment is available at HumanConnectome.org, from the Human Connectome Project (HCP) (Van Essen et al., 2013). The bundle atlas is available at zenodo.org/record/3613688 (Garyfallidis et al., 2018; Yeh et al., 2018). 

Resulting streamlines generated during the current study are available from the corresponding author on reasonable request. Where both the tractography algorithm and RecoBundles segmentation were computed with *Dipy* at dipy.org (Garyfallidis et al., 2014). An open source implementation of the proposed *Fast Streamline Search* is available in Dipy.

## Appendix

### A. Sum of Norm Properties with Detailed Equations

#### Proof (Remark 1)

The dist$_{L^1}(U,W)$ is equivalent to computing the $L^1$ distance between two $m \times n$ dimensional points ($\mathbf{u }, \mathbf{w } \in \mathbb {R}^{m \times n}$).

$$\begin{aligned} \text {dist}_{L^1}(U,W)&= \sum _{i=1}^m || \mathbf{u} _i - \mathbf{w} _i ||_1 \\&=\sum _{i=1}^m \sum _{j=1}^n |u_{i,j}-w_{i,j}| \\&= || \mathbf{u } - \mathbf{w } ||_1 \\ \end{aligned}$$

$$\square$$

#### Proof (Remark 2)

The $L^1$ distance in *n*-dimensions can be used as an upper and a lower bound the $L^2$ distance, from Hölder’s inequality ($\mathbf{x} \in \mathbb {R}^n$).

$$\begin{aligned} \left\| \mathbf{x} \right\| _p \le&\left\| \mathbf{x} \right\| _r \le n^{ (1/r - 1/p) } \left\| \mathbf{x} \right\| _p&0< r < p \\ \left\| \mathbf{x} \right\| _2 \le&\left\| \mathbf{x} \right\| _1 \le \sqrt{n} \left\| \mathbf{x} \right\| _2&r=1,\, p=2 \\ \frac{1}{\sqrt{n}}\left\| \mathbf{x} \right\| _2 \le&\frac{1}{\sqrt{n}}\left\| \mathbf{x} \right\| _1 \le \left\| \mathbf{x} \right\| _2&\text {division by } \frac{1}{\sqrt{n}} \end{aligned}$$

$$\square$$

#### Proof (Remark 3)

The distance between the mean position ($\overline{\mathbf{u }} = \frac{1}{m} \sum _{i=1}^m \mathbf{u} _i$) of two streamlines is always smaller or equal to the average point-wise distance. This can be obtained from $L^p$-norm properties $(1 \le p < \infty \,,\; \mathbf{x} ,\mathbf{y} \in \mathbb {R}^n \,,\; \lambda \in \mathbb {R})$.

11 $$\begin{aligned} \left\| \mathbf{x} + \mathbf{y} \right\| _p \,\le \, \left\| \mathbf{x} \right\| _p + \left\| \mathbf{y} \right\| _p \text {triangle inequality} \end{aligned}$$

12 $$\begin{aligned} \left\| \lambda \mathbf{x} \right\| _p \,=\, |\lambda | \, \left\| \mathbf{y} \right\| _p \text {positive homogeneity} \end{aligned}$$

Using $\mathbf{u} _i,\mathbf{w} _i \in {\mathbb{R}}^n ,\, i \in \{1, ..., m\}$, such that $\mathbf{d} _i = \mathbf{u} _i - \mathbf{w} _i$

$$\begin{aligned} \big \Vert \overline{\mathbf{u }} - \overline{\mathbf{w }} \big \Vert _p&= \bigg \Vert \, \frac{1}{m}\sum _{i=1}^m \mathbf{u} _i - \frac{1}{m} \sum _{i=1}^m\mathbf{w} _i \,\bigg \Vert _p \\&= \frac{1}{m}\bigg \Vert \, \sum _{i=1}^m (\mathbf{u} _i - \mathbf{w} _i) \,\bigg \Vert _p&\text {from (12)}\\&\le \frac{1}{m} \sum _{i=1}^m \big \Vert \mathbf{u} _i - \mathbf{w} _i \big \Vert _p&\text {from (11)} \end{aligned}$$

$$\square$$  
